# Influence spreading model used to analyse social networks and detect sub-communities

**DOI:** 10.1186/s40649-018-0060-z

**Published:** 2018-11-29

**Authors:** Vesa Kuikka

**Affiliations:** Finnish Defence Research Agency, PO BOX 10, Tykkikentäntie 1, 11311 Riihimäki, Finland

**Keywords:** Social networks, Influence spreading, Network dynamics, Influence measure, Network topology, Community detection, Community structure, Closeness centrality, Betweenness centrality

## Abstract

A dynamic influence spreading model is presented for computing network centrality and betweenness measures. Network topology, and possible directed connections and unequal weights of nodes and links, are essential features of the model. The same influence spreading model is used for community detection in social networks and for analysis of network structures. Weaker connections give rise to more sub-communities whereas stronger ties increase the cohesion of a community. The validity of the method is demonstrated with different social networks. Our model takes into account different paths between nodes in the network structure. The dependency of different paths having common links at the beginning of their paths makes the model more realistic compared to classical structural, simulation and random walk models. The influence of all nodes in a network has not been satisfactorily understood. Existing models may underestimate the spreading power of interconnected peripheral nodes as initiators of dynamic processes in social, biological and technical networks.

## Background

Social influence measures have been developed by using for example local structural characteristics [1, 2] geodesic distances [3] and random walks [4]. Most of these measures don’t have exact quantitative interpretations for general network structures and variable sizes of networks. Structural measures take into account local degrees of nodes in the neighbourhood of a source node. Geodesic based measures use distances from a source node. Random walks consider different paths from a source node to a target node but the method still is unsuccessful in combining the contributions from alternative paths to generate an exact quantitative measure.

Models for the process by which influence or ideas propagate through a social network have been studied in a number of research articles for example in [5–9]. In a recent article [10] a review of theories for influencer identification in complex networks has been published. Many aspects should be considered when constructing measures for describing and comparing social networks. Several studies propose influence measures for identifying the most influential spreaders or mediators. Obviously when spreading processes are analysed the concept of time should have some kind of role in the model. Some models presented in the literature are static and don’t investigate processes evolving dynamically or don’t provide justification for how the models describe a steady state or limiting states of a network. Usually network structures are not calculated exactly random walk in a network is an example. One requirement for the theory is a quantitative model with natural interpretations of the variables. This guarantees that the numerical values obtained for all kinds of network topologies and different temporal spreading distributions can be compared with each other. A valid theory and an applicable model are needed to combine the spreading process evolving as a function of time and the structure of a network.

Computational difficulties must be solved in keeping track of various paths and their possible interdependencies. In large networks computing time may set practical constraints for calculations. One requirement for research of large social biological and technical networks is a scalable computing algorithm [11]. Good approximations can be achieved with limited path lengths as the rule of six degrees [12] is valid for many kinds of social networks. Limited path lengths can provide good results in community detection algorithms. In the literature many community detection algorithms take into account only local interactions [13].

## Introduction

The aim of this paper is to provide answers to the requirements presented in the previous section. Possible models for describing the temporal spreading process are proposed. A method for modelling the topological structure of a network is presented. Probability theory is used for combining the spreading via all the possible paths from a source node to a target node. Possible dependencies between different paths are taken into account. With these building blocks various problems in social network analysis, and in many other fields of network science, can be solved [14–16].

We present specifications for the most important measures needed to investigate social networks. These are node level ego centric centrality and betweenness measures. Closeness centrality describes node’s power to spread influence to other nodes in the network. Betweenness is a measure of the influence of nodes in a network relative to the flow of information between others. Betweenness centrality tends to pick out nodes that play the role of brokers between communities. In addition, an overall network measure, expressed as a function of time that combines different properties of the network, is presented. After all, different measures for different purposes can be constructed. For example, the concept of betweenness can be understood in many ways which makes it impossible to define one absolute betweenness measure.

We demonstrate the method with a real social network documented in the literature and compare the results with the corresponding study published recently. The same network has been investigated in [3] where a comprehensive model suitable for local and global aspects of a social network has been presented. In [3], a model with an adjustable parameter for weighting neighbouring and distant nodes in the network has been used to determine measures for centrality and betweenness.

In many networks a community structure exists, in which network nodes are connected together in groups, between which there are less connections. A number of methods and algorithms have been proposed for detecting communities in social networks, for example published in [17–23]. The research has often been focused on developing different or more efficient algorithms and different implicit or explicit definitions of community [24, 25]. As different definitions of community exist, different algorithms are needed for discovering various kinds of communities.

Some of the algorithms for detecting communities in a network structure are minimum-cut method, hierarchical clustering, Girvan–Newman algorithm, modularity maximization, statistical inference [26], and clique-based methods. Descriptions of the methods can be found for example in [13, 24, 25, 27]. Many classical algorithms for partitioning network nodes into groups are based on matrix and linear algebra methods. Examples are analogues of the Kernighan–Lin algorithm [28] for maximizing modularity and an analogue of the spectral graph partitioning [29, 30] algorithms for community detection. A definition for modularity is the fraction of the edges that fall within the given group minus the expected fraction if edges were distributed at random. The Kernighan–Lin algorithm is based on repeatedly moving, starting from some initial division, the vertices that most increase or least decrease the modularity.

The Louvain method for community detection is a greedy modularity optimization method to extract communities from large networks [31]. For investigation of large-scale biological and social community structures an information theoretic approach has been presented in [32]. Probability flow of random walks on a network is used as a proxy for information flows. There are a number of other greedy or SDP-based (semi-definite programming) approaches for finding communities in large networks [33, 34].

A classification for community discovery methods in complex networks has been presented in [24]. Eight different community discovery methods have been described in the review: feature distance, internal density, bridge detection, diffusion, closeness, structure, link clustering and meta-clustering. Altogether 39 algorithms classified in these eight categories have been described in [24]. One of the methods is more relevant from our perspective: a diffusion community in a complex network is a set of nodes that are grouped together by the propagation of the same property, action or information in the network. In [24] a meta-procedure for detecting a diffusion community has been defined: Perform a diffusion or percolation procedure on the network following a particular set of transmission rules and then group together any nodes that end up in the same state. In this respect, a community can be defined as a set of target nodes influenced by a fixed set of source nodes. In the financial networks literature, a decaying influence model describing propagation of shocks on banking networks has been studied in [35].

## Outline

The focus of this paper is to present a new influence spreading model and its applications with examples. Accordingly, the main content of this study is presented in “Theory of social influence measures”, “Applications of social influence measures” and “Numerical results and discussion” sections. In addition, the next section introduces classical definitions of closeness centrality and betweenness centrality as well a recent extension of the measures to consider both local and global network structure. Lastly, conclusions provide a short summary of the paper.

The theory is presented in several phases. First, information and influence propagation models are discussed. Next, the influence spreading measure between two nodes of a network is presented with the help of an example network of Dutch students’ social network [3, 36]. Then follow definitions of quantities and the general method of combining paths between two nodes of a network. Temporal Spreading of Influence is a sub-model describing time dependence of the spreading process. After this, a high-level algorithm is presented for computing the influence spreading matrix describing the spreading between all nodes of a network.

Applications of the theory of Social Influence Measures are based on the Social Influence Matrix. In this part of the study, definitions of closeness centrality, betweenness centrality and community detection measures are presented.

The model is demonstrated by presenting results for closeness centrality, betweenness centrality and analysis of community structures. Closeness centrality and betweenness centrality measures are illustrated with the Dutch students’ social network [3, 36]. Four different networks are used as examples for detecting communities and investigating network structures. As an introduction, an artificial network of the Game of Risk [37] is analysed. Then the 32 Dutch students’ social network [36] is investigated introducing more complex structures. Next, an animal social network of dolphins [38] is analysed along with some comments on similarities and differences with respect to human social networks. The scalable version of the algorithm [11] is used for computing the influence spreading matrix for a Facebook social network of 4039 users. The matrix is used as input information for the community detection algorithm.

## Geodesic based centrality and betweenness measures

Several measures of centrality and betweenness have been proposed in the literature [1, 39]. Recently, geodesic based centrality and betweenness measures, unifying the local and the global network structure, have been presented [3].

A normalized version of reciprocal closeness centrality [3, 16] is defined by

1 $$C_{\text{C}} \left( i \right) = \frac{{\mathop \sum \nolimits_{j \ne i} \left( {g_{ij} } \right)^{ - 1} }}{N - 1},$$

where the geodesic distance $g_{ij}$ is the distance between ego $i$ and all its others $j$. *N* is the total number of nodes in the network. In [3] a generalization of Eq. (1) has been proposed that weights nodes at different distances depending on the value of a gradient parameter $\delta$:

2 $$C_{C}^{\delta } \left( i \right) = \frac{{\mathop \sum \nolimits_{j \ne i} \left( {g_{ij} } \right)^{ - \delta } }}{N - 1},$$

where $$\delta \ge 0.$$

Classical betweenness centrality measure focuses on the power resulting from being on the shortest path among others. A node with high betweenness centrality is a broker between others in the network. This involves three actors, with the focus on actor $i$ being on the shortest path between actors $j$ and $k$. Let $t_{jk}$ denote the total number of shortest paths connecting $j$ to $k$ and $t_{jik}$ be the number of shortest paths connecting $j$ to $k$ that pass through $i$ then the betweenness of $i$ [3] is defined by

$$C_{\text{B}} \left( i \right) = \mathop \sum \limits_{j < k} \left( {\frac{{t_{jik} }}{{t_{jk} }}} \right).$$

Again, in [3] a generalization has been proposed depending on the value of a gradient parameter $\delta$:

$$C_{B}^{\delta } \left( i \right) = \mathop \sum \limits_{j < k} \left( {\frac{{t_{jik} }}{{t_{jk} }}} \right)\left( {g_{jk} - 1} \right)^{ - \delta } .$$

## Theory of social influence measures

### Information and influence propagation models

Different propagation models for influence spreading can be defined depending on the phenomena we are studying. In the context of this paper, two main issues are important. Firstly, a model has to be decided for the time distribution that describes spreading of influence from one node to another. Secondly, propagation can proceed independently of states of mediating nodes, or propagation depends on the states of the nodes along the paths between a source and a target node.

In this paper we use Poisson distribution as the time distribution for propagation between nodes. In the model, it is easy to use any statistical distribution or empirical data instead of Poisson distribution. We have made experiments with a model based on Uniform distribution. This describes, for example, propagation of information via e-mails when users process their e-mails at uniformly distributed time points during a day (or other time unit). This distribution gives comparable results, but not exactly the same, because more spreading occurs at low time values when propagation obeys Uniform instead of Poisson distribution.

The second issue, when the spreading process depends on the states of the intermediate nodes, is more involved. Dependency on static node attributes is a minor addition to the model because the model takes into account nodes and links individually. Dynamic dependency on time dependent states of nodes can be compute-intensive because simulations or iterative algorithms probably are necessary to solve the problem. In this paper, only state independent propagation models are studied. Nodes mediate influence regardless of their own state and states of all the other nodes of the network.

A realistic model for information propagation may be a state dependent variant of the model where propagation events (attempts of influence) occur only for new information (or probability for new information is higher). In other words, information is mediated to neighbouring nodes only in cases when the node is unaware of the information before the propagation event. Nodes are less willing to propagate known information than new information.

Propaganda, or other form of influence, transforms its content during the spreading process. Therefore, recurrent propagation events are more realistic. However, decreasing in amount as a function of path length limits the process. In the model of this paper, this is accomplished with combined effects of time dependency and node (and link) weighting factors. Weighting factors, that are less than one, are realistic when nodes are not fully actively propagating influence. In summary, state dependent and state independent alternatives are the following:

1. Probability of spreading influence depends on the states of nodes. Nodes along the paths between a source and a target typically are less eager to mediate information already known.
2. In the state independent model, propagation occurs independently of nodes’ states. The probability to receive and forward influence is determined by the time dependent probability, node weighting factor and link weighting factor.

In our model nodes are assumed to be memoryless. Receiving an attempt to influence node’s state and propagating this event forward are assumed to have delays according to the temporal distribution, e.g. Poisson distribution.

1. In summary, the propagation model has the following characteristics: temporal distribution describes node’s delays between receiving an influence spreading event and forwarding the event to neighbouring nodes. Links between nodes have no delays.
2. Node weighting factor $w_{N = i}$ for node $i$ describes node $i$’s activity, that is, the probability of forwarding an event of influence to neighbouring nodes. Similarly, link weighting factors $w_{L = i,j}$ are additional factors needed in cases where the influence spreading between nodes $i$ and $j$ are not equal for all the directed links between nodes of the network.
3. The spreading process is assumed to start from one node in the network at time $T = 0$.

## Influence spreading measure between two nodes

### Example network

In this section, we illustrate mathematical methods of modelling influence spreading measures $C_{s,t} \left( T \right)$ between a source node $s$ and a target node $t$ in a small social network at time $T$. Based on these results, new measures of centrality, betweenness and community detection are defined.

**Fig. 1:**
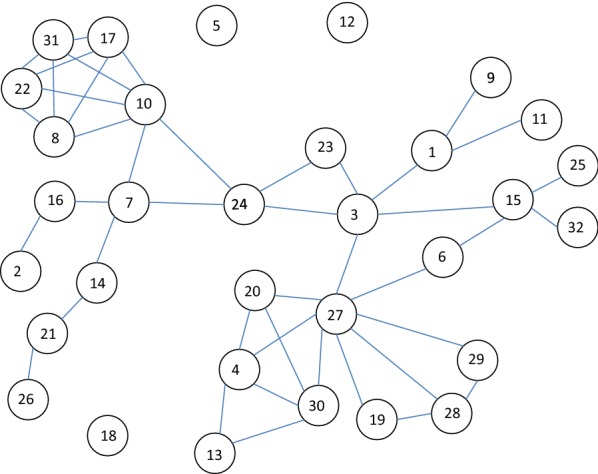
Symmetrized friendship network among 32 Dutch students [36]

The method aims at solving the requirements explained in the “Background” section. As the recent study in [3] has similarities and many common objectives, we use the same social network of 32 Dutch students [36]. This gives us the possibility to compare the numerical results between the two models. The network is shown in Fig. 1.

Our method takes into account all the possible self-avoiding paths in the network. The generalization including paths allowing nodes to appear several times in a path is possible and easy to compute [11]. However, then we must have a limit for path lengths or for the number of possible occurrences on the path. This method requires less computer memory because the list paths need not to be saved in computer memory. For large networks the number of different paths is high and saving memory is important. Self-avoiding paths are suitable for the purposes of presenting the method. Later in this paper, results for a larger social network of 4039 Facebook users will be provided where influence propagation via paths with loops is considered.

**Fig. 2:**
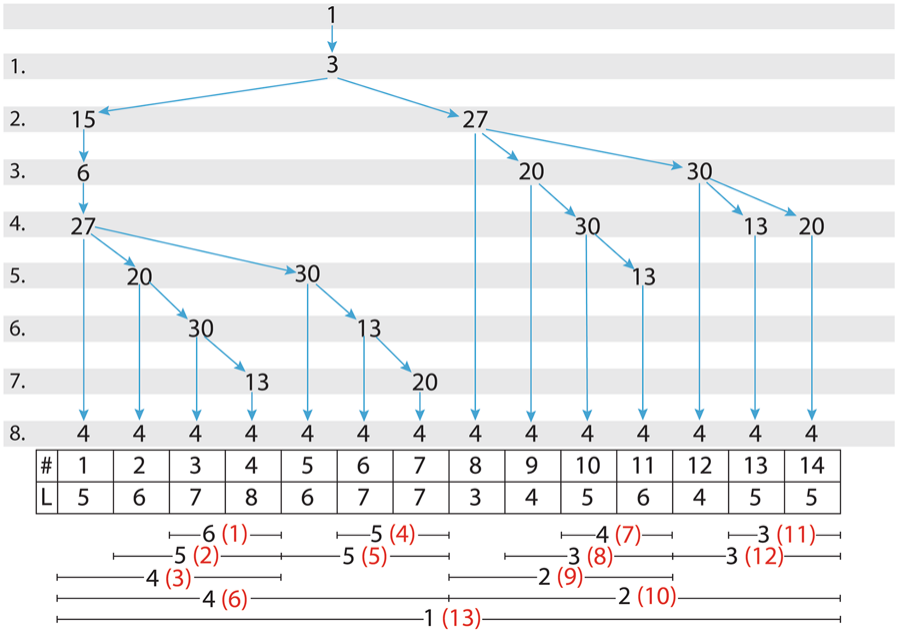
The 14 paths from Node 1 to Node 4 presented as a hierarchical tree. Nodes of the paths are shown in the tree. The first line (#) indicates the running number of the paths and the second line (*L*) shows the path lengths. In the lower part of the figure the order of calculation is shown in parenthesis, after a number giving the length of common paths in combing the paths, indicated by the line segments

As an example, all the self-avoiding paths of the network of Fig. 1 from Node 1 to Node 4 are listed Table 1. As all the paths pass through Node 3, all the 14 paths have dependencies with each other. They have the common link 1–3 from Node 1 to Node 3. The same procedure of finding common paths at the beginning of different paths originating from a source node to a target node is used iteratively. Figure 2 shows the paths of Table 1 in another format.

**Table 1:** The 14 paths from Node 1 to Node 4 of the network in Fig. 1

#	Nodes in a path								
1	1	3	15	6	27	4			
2	1	3	15	6	27	20	4		
3	1	3	15	6	27	20	30	4	
4	1	3	15	6	27	20	30	13	4
5	1	3	15	6	27	30	4		
6	1	3	15	6	27	30	13	4	
7	1	3	15	6	27	30	20	4	
8	1	3	27	4					
9	1	3	27	20	4				
10	1	3	27	20	30	4			
11	1	3	27	20	30	13	4		
12	1	3	27	30	4				
13	1	3	27	30	13	4			
14	1	3	27	30	20	4

To be precise, we present below in Eq. (4) all the steps of computing the probability of influence spreading from Node 1 to Node 4. We denote intermediate steps by subscripts in parentheses. For example, in the first step, $P_{\left( 1 \right)}$ is the combined result of paths 1-3-15-6-27-20-30-4 and 1-3-15-6-27-20-30-13-4 with path lengths 7 and 8 and common path length 6. The steps of the computing algorithm are shown in parenthesis in Fig. 2. The last step in Eq. (4) denoted by $G_{1,4,\left( 1 \right)}$ gives the final result of the influence of Node 1 on Node 4.

In the following, we use the short hand notations $C_{6}$ = 1-3-15-6-27-20-30, $B_{1}$ = 30-4, and $B_{2}$ = 30-13-4 and denote the conditional probabilities by $P_{1} (B_{1} |C_{6} )$ and $P_{2} (B_{2} |C_{6} )$. Also, time variable $T$ is omitted. For example, $P_{1} (B_{1} |C_{6} )$ is the probability that the spreading goes through one link more (path $B_{1}$) after the spreading has already propagated through six links (path $C_{6}$). The justification for the first equation $P_{\left( 1 \right)}$ follows from the following probabilistic formula:

3 $$\begin{aligned} P_{(1)} & = P_{6} (C_{6} )[P_{1} (B_{1} |C_{6} ) + P_{2} (B_{2} |C_{6} ) - P_{1} (B_{1} |C_{6} )P_{2} (B_{2} |C_{6} )] \\ & = P_{6} (C_{6} )P_{1} (B_{1} |C_{6} ) + P_{6} (C_{6} )P_{2} (B_{2} |C_{6} ) - \frac{{P_{6} (C_{6} )P_{1} (B_{1} |C_{6} )P_{6} (C_{6} )P_{2} (B_{2} |C_{6} )}}{{P_{6} (C_{6} )}}. \\ \end{aligned}$$

Below, all the intermediate steps of computing the influence of Node 1 on Node 4 are shown. We present only one example of the algorithm as this makes it possible to write the equations for all the connections between all the nodes in a network. We have developed a computer programme for finding all the possible paths and computing the probabilities according to the theory. In the programme, maximum path lengths can be used to limit the computing time. For typical small social networks presented in the literature, there is no need to limit the path lengths. In larger networks the results converge rapidly and a reasonable limit (for example, path lengths between 6 and 10) can be used to get good approximations.

4 $$\begin{array}{l} P_{(1)} = P_{7} + P_{8} - \frac{{P_{7} P_{8} }}{{P_{6} }} \qquad P_{(2)} = P_{(1)} + P_{6} - \frac{{P_{(1)} P_{6} }}{{P_{5} }} \\ P_{(3)} = P_{(2)} + P_{5} - \frac{{P_{(2)} P_{5} }}{{P_{4} }} \quad P_{(4)} = P_{7} + P_{7} - \frac{{P_{7} P_{7} }}{{P_{5} }} \\ P_{(5)} = P_{6} + P_{(4)} - \frac{{P_{6} P_{(4)} }}{{P_{5} }}\quad P_{(6)} = P_{(3)} + P_{(5)} - \frac{{P_{(3)} P_{(5)} }}{{P_{4} }} \\ P_{(7)} = P_{5} + P_{6} - \frac{{P_{5} P_{6} }}{{P_{4} }}\qquad P_{(8)} = P_{4} + P_{(7)} - \frac{{P_{4} P_{(7)} }}{{P_{3} }} \\ P_{(9)} = P_{3} + P_{(8)} - \frac{{P_{3} P_{(8)} }}{{P_{2} }}\quad P_{(10)} = P_{(9)} + P_{(12)} - \frac{{P_{(9)} P_{(12)} }}{{P_{2} }} \\ P_{(11)} = P_{5} + P_{5} - \frac{{P_{5} P_{5} }}{{P_{3} }}\quad \; P_{(12)} = P_{4} + P_{(11)} - \frac{{P_{4} P_{(11)} }}{{P_{3} }} \\ G_{1,4,(1)} = P_{(13)} = P_{(6)} + P_{(10)} - \frac{{P_{(6)} P_{(10)} }}{{P_{1} }} \\ \end{array}$$

In the example of computing the influence of Node 1 on Node 4 all the paths go through Node 3 (link 1–3). If we consider the influence of Node 3 on Node 4, we observe from Fig. 1 that two independent possibilities occur, via links 3–27 and 3–15–6–27. In this particular case, these two contributions are denoted by $G_{3,4,\left( 1 \right)}$ and $G_{3,4,\left( 2 \right)}$. In the following, we denote the number of possible independent contributions by ${\mathcal{I}}$.

### Combining paths between two nodes

Next, we present the general formulation of the theory. In Eq. (5) $G_{n,j,\left( x \right)} \left( {w,T} \right), \quad x = 1, \ldots ,{\mathcal{I}}$ describe independent contributions computed with the algorithm. Combining all the independent contributions of Node *n* on Node *j* we get:

5 $$C_{n,j} \left( {w,T} \right) = 1 - \mathop \prod \limits_{x = 1}^{{\mathcal{I}}} \left( {1 - G_{n,j,\left( x \right)} \left( {w,T} \right)} \right), \quad n,j = 1, \ldots ,N ,$$

where $G_{n,j,\left( x \right)} \left( {w,T} \right)$ is the probability of spreading from Node $n$ to Node $j$ via Link ($x$), where ($x$) denotes an index of the ${\mathcal{I}}$ links originating form Node $n$ (degree of Node $n$) at time $T$. In Eq. (5) node and link weighting factors along the path from Node $n$ to Node $j$ are denoted by vector $w = \left( {w_{N} ,w_{L} } \right)$ (see Eq. 6), and $N$ is the number of nodes in the network.

Computing $G_{n,j,\left( x \right)} \left( {w,T} \right)$ requires searching all the different paths from Node $n$ to Node $j$ with path lengths less than an upper limit ${L_\text{max} }$. Parameter $L_{ \text{max} }$ is the maximum path length and it is used to restrict the number of paths and computing time in large networks. Searching the paths is a straightforward task by using the network topology information by following links between the source node and target nodes. The computation is conducted simultaneously from one source node to all the nodes in the network. The algorithm for computing $G_{n,j,\left( x \right)} \left( {w,T} \right)$ handles the paths in the descending order of the number of common links at their beginning among the set of paths from Node $n$ to Node $j$. A simple method would be first to list all the paths and then compute the influence spreading matrix $C_{n,j} \left( {w,T} \right), \quad n,j = 1, \ldots ,N$.

The most time consuming task, when computing self-avoiding paths, is keeping track of nodes and rejecting paths where a node appears more than once. This is the reason why the algorithm relaxing the condition of self-avoidance and allowing loops has significantly lower computer running times, essential for large social networks [11].

Weighting factors describe probability of propagating information and opinions. We call this the activity of nodes (or links). Opinion changes in social networks are uncommon when the new ideas are unfamiliar to members of a social network. (To be precise, we should make a difference between influence spreading and opinion spreading. These concepts are related but usually different parameters are needed. Even a different spreading model may be needed, if probability to change opinion is conditional on information or influence spreading events). Technical and biological networks have similar commonalities. Spreading of a computer virus or a biological virus between nodes can have a low probability because of virus protection, vaccination or characteristics of the virus itself.

We illustrate the propagation rules with an example of combining two paths. Also, the effects of node and link weighting factors are shown explicitly. In the algorithm any number of paths can be combined iteratively by using the same method. Combining the effects of different paths between two nodes is computed in the descending order of common path lengths of paths starting from the initial node. Only these common links and nodes are taken into account. If the paths join later or cross each other, they are considered independent events. The probability of influence spreading from Node $s$ to Node $t$ via path of lengths $L_{1}$ is

6 $$C_{s,t} \left( T \right) = w_{N = t} W_{{L_{1} }} D_{{L_{1} }} \left( T \right),$$

where

$$W_{{L_{1} }} = \mathop \prod \limits_{j = 0}^{{L_{1} - 1}} w_{N = I\left( j \right)} w_{{L = I\left( j \right),I\left( {j + 1} \right)}} ,$$

where $w_{N}$ are node weighting factors, $w_{L}$ are link weighting factors, and $D_{L} \left( T \right)$ is the time dependence of influence spreading process (see $D_{L} \left( T \right)$ in Eq. (8) for Poisson distribution). Function $I\left( j \right),\quad j = 0, \ldots ,L_{1}$ maps index $j$, describing the order of nodes on the path from Node $s$ to Node $t$, to the unique indexing $\left\{ {1, \ldots ,N} \right\}$ of all nodes in the network. For example, $s = I\left( 0 \right)$ and $t = I\left( {L_{1} } \right)$. In our calculations we will use for the first node the activity value of $w_{s} = 1$. The first node initiates the influence propagation process at time $T = 0$. $D_{{L_{1} }} \left( T \right)$ is the probability of influence propagation via single path length $L_{1}$ during time interval $\left[ {0,T} \right]$. Note that in Eq. (6) node and link weights are not included in $D_{{L_{1} }} \left( T \right)$.

$D_{{L_{1} }} \left( T \right)$ can be expressed as $D_{{L_{1} }} \left( T \right) = D_{L} \left( T \right)D_{{L_{1} - L}} \left( T \right)$, where $D_{{L_{1} - L}} \left( T \right)$ is the conditional probability of forwarding an influence spreading event via path length $L_{1} - L$, given that the event has passed via path length $L$ before that during $\left[ {0,T} \right]$. Similarly, $W_{{L_{1} }}$ can be expressed as $W_{{L_{1} }} = W_{L} w_{m} W_{{L_{1} - L}}$, where $w_{m}$ is the node weighting factor of the last node $m$ of the path of length $L$. Next we assume that influence events can propagate via two routes of lengths $L_{1}$ and $L_{2}$ with a common path of length $L$ at their beginning. If the paths join later, we assume that they are independent attempts of influence. In the model, we get for the probability of influence spreading via the two routes:

7 $$\begin{aligned} C_{s,t} \left( T \right) & = w_{t} w_{m} W_{L} D_{L} \left( T \right)\left( {W_{{L_{1} - L}} D_{{L_{1} - L}} \left( T \right) + W_{{L_{2} - L}} D_{{L_{2} - L}} \left( T \right) - W_{{L_{1} - L}} D_{{L_{1} - L}} \left( T \right)W_{{L_{2} - L}} D_{{L_{2} - L}} \left( T \right)} \right) \\ & = P_{{L_{1} }} \left( T \right) + P_{{L_{2} }} \left( T \right) - \frac{{P_{{L_{1} }} \left( T \right)P_{{L_{2} }} \left( T \right)}}{{P_{L} \left( T \right)}}, \\ \end{aligned}$$

where a shorter notation $P_{{L_{i} }} \left( T \right)$ is used for $w_{t} W_{{L_{i} }} D_{{L_{i} }} \left( T \right)$ describing the probability of influence propagation over the path of length $L_{i}$. In following sections, the algorithm is demonstrated with a more general example of a real-life social network.

At the beginning of this section, Eq. (5) describes non-mutually exclusive events in basic probability theory. It serves as an introduction between commonly known methods of probability and the method of this study for combining probabilities of influence spreading via different paths in a network. In fact, Eq. (5) is the last step in the algorithm with $L = 0$ and $P_{L} \left( T \right) = 1$ in Eq. (7). As a consequence, we could have omitted Eq. (5) because it can be regarded as the last step of the general algorithm.

### Temporal spreading distribution

Before we can compute numerically the contributions of different paths of a network, we must have a model for the probabilities $D_{L} \left( T \right)$ of temporal spreading on a chain of nodes. The number of links from a source node to a target node (path length) is denoted by $L$. Assuming Poisson distribution the probability of at least $L$ events occurring is:

8 $$D_{L} \left( T \right) = P\left( {K\left( T \right) \ge L} \right) = 1 - \mathop \sum \limits_{z = 0}^{L - 1} e^{ - \lambda T} \frac{{\left( {\lambda T} \right)}}{z!}, \left( {D_{0} = 1} \right).$$

Here, the interpretation is that the spreading has advanced $L$ or more links in the network at time $T$. Equation (8) takes into account nodes’ delays between receiving an influence spreading event and forwarding the event to neighbouring nodes. When time approaches infinity, nodes’ probability of spreading influence approaches one. In Eq. (8), the number of spreading events is denoted by stochastic variable $K\left( T \right)$. The intensity parameter of Poisson distribution is denoted by $\lambda .$ The statistical distribution and its parameters determine the spreading rate in the network. The Poisson distribution is not the only possibility, for example, a model based on Uniform distribution may better describe some other temporal spreading behaviour.

Parameter $\lambda$ can be estimated from empirical influence propagation data. In most cases, this kind of time dependent information is not available. If empirical data are not available, the intensity parameter could be evaluated by comparing with analyses of other networks with comparable level of development. Values of $\lambda$ and time $T$ are related in Eq. (8), and also the quantity $\lambda T$ can be estimated. It describes the maturity level of propagation on the network. In practice, evaluating the model parameters is not simple because nodes may have individual characteristics. But if these kind of empirical data are available, the model can be used with different parameters for each node and link of the network. In addition, the stochastic distribution of Eq. (8) can be replaced by an empirical distribution.

### Algorithm for computing the influence spreading matrix

An algorithm for computing the values of bidirectional influence measures [11] between all the pairs of nodes in a network is presented. These values make up a $N \times N$ dimensional influence spreading matrix, were $N$ is the number of nodes in the network. The matrix is computed for discrete spreading time values of interest. Closeness centrality, betweenness centrality and community detection measure are defined with the help of the matrix elements. The algorithm for computing the influence spreading matrix $C_{s,t} \left( T \right), \quad s,t = 1, \ldots ,N$ is described below. Comments in the algorithm below are denoted by ‘/* */’.


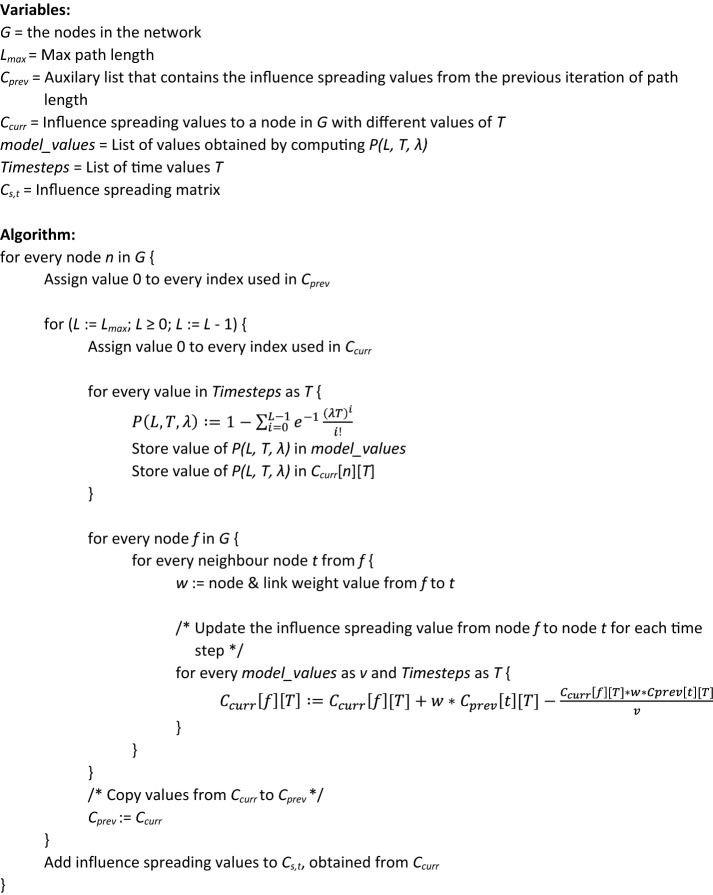


The applicability of shortest-path-based centralities is limited by the high computational complexity of calculating the shortest paths between all pairs of nodes [10]. A generalization of closeness centrality [40] considers all paths in the network and assigns a larger weight to shorter paths using a tuneable parameter. The method presented in this paper considers all the paths in a network with an additional feature of modelling common links of the paths at the beginning of their routes from a source node to target nodes.

Most of the results of this paper are for self-avoiding paths. A self-avoiding path is a sequence of moves on a path that does not visit the same node more than once. The networks of this paper are selected for illustrative purposes and therefore are small when compared with many modern applications and interests of research.

With this in mind, a fast algorithm has been developed for analysing large social networks. In [11] large scale social networks of Facebook, Twitter and Google+ have been investigated. Computing times with a PC hardware (Core2 Duo E7503) of closeness centralities for all the nodes in the networks are shown in Table 2.

**Table 2:** Computing times of the fast algorithm [11] for large social media networks

Social media	Nodes	Links	Computing time
Facebook	4039	88,234	1 min
Twitter	81,306	1,768,149	5 h
Google+	107,614	13,673,453	4 days

The algorithm, especially suitable for influence spreading modelling of social networks, allows returns to nodes during the spreading process. The design of the algorithm is based on this property. In fact, disallowing loops would make the algorithm in [11] slower. The value of maximum path length $L_{ \text{max} }$ can be set to 20 because higher terms are negligible for typical temporal spreading distributions. Lower values are used if they describe the real-world phenomenon by limiting the spreading process to shorter path lengths.

## Applications of social influence measures

### Definition of closeness centrality measures

In the following we use both normalized and un-normalized versions of centrality and betweenness measures. Normalized measures are divided by the number of nodes $N$ of the network. These measures have a natural interpretation, normalized measures are probabilities and un-normalized measures are expressed in the units of number of nodes.

Equations (9), (10) and (11) are proposed measures for source centrality, target centrality and betweenness correspondingly. Usually, these measures are highly correlated, for example, Eqs. (9) and (10) can be regarded as two different viewpoints of node’s centrality in the network. The measure in Eq. (9) has the summation over target nodes instead of the summation over source nodes in Eq. (10). The interpretation of Eq. (9) is a measure of influence of Node $n$ on all other nodes in the network. The measure of Eq. (10) describes the influence of all the nodes of the network on Node $m$.

9 $$\frac{{C_{n, \cdot } \left( {w,T} \right)}}{N} = c_{n, \cdot } \left( {w,T} \right) = \frac{1}{N}\sum\limits_{j = 1}^{N} {C_{n,j} \left( {w,T} \right)}$$

10 $$\frac{{C_{ \cdot ,m} \left( {w,T} \right)}}{N} = c_{ \cdot ,m} \left( {w,T} \right) = \frac{1}{N}\sum\limits_{i = 1}^{N} {C_{i,m} \left( {w,T} \right)}$$

In Eqs. (9, 10) $C_{i,j}$ is defined in Eq. (5) or equivalently in Eq. (7) and $T$ is time. In the next sections, the measure in Eq. (9) is regarded as the default viewpoint and we denote $C_{n, \cdot }$ by $C_{n}$ as a short hand notation. From Eqs. (9, 10) a cohesion measure describing the two aspects of these equations can be defined:

11 $$\frac{{C\left( {w,T} \right)}}{N} = c\left( {w,T} \right) = \frac{1}{N}\sum\limits_{i = 1}^{N} {c_{i, \cdot } } \left( {w,T} \right) = \frac{1}{N}\sum\limits_{j = 1}^{N} {c_{ \cdot ,j} \left( {w,T} \right)}$$

### Definition of betweenness centrality measures

The idea of defining betweenness measures is based on removing one node form the network. In Eq. (12) Node $n$ is removed from the network and after that the betweenness measure for Node $n$ is calculated in a consistent way with Eqs. (9, 10). We denote $n \notin V$ indicating that Node $n$ is removed from the network. Note that any order of summations in Eq. (12) provides the same results. This is a desirable feature of a betweenness measure. In other words, source nodes and target nodes are in a symmetric position. The measure of Eq. (12) describes the betweenness of Node $n$ in the network.

12 $$\frac{{B_{n} \left( {w,T} \right)}}{N} = \frac{1}{{N^{2} }}\mathop \sum \limits_{{\begin{array}{*{20}c} {i = 1} \\ {n \notin V} \\ \end{array} }}^{N} \mathop \sum \limits_{{\begin{array}{*{20}c} {j = 1} \\ {n \notin V} \\ \end{array} }}^{N} \left( {1 - \mathop \prod \limits_{x = 1}^{{\mathcal{J}}} \left( {1 - G_{i,j,\left( x \right)} \left( {w,T} \right)} \right)} \right)$$

In the definition of Eqs. (9–12) normalization is a question. We have decided to include source nodes with the value of 1.0 in the formulas and as a result of that *N* is used as a normalization factor. The source node is assumed to be the initiator of influence spreading with probability 1.0.

We can define another measure by dividing Eq. (12) by Eq. (11). This ratio gives the proportional quantity of Eq. (13):

13 $$R_{n} \left( {w,T} \right) = \frac{{B_{n} \left( {w,T} \right)}}{{C\left( {w,T} \right)}}$$

Both Eqs. (12) and (13) preserve the same rankings of nodes in the network. The interpretation is that the lowest curve has the highest betweenness. Further, we define a betweenness centrality measure with the help of Eq. (12) as

14 $$b_{n} = 1 - R_{n} \left( {w,T} \right) = \frac{{C\left( {w,T} \right) - B_{n} \left( {w,T} \right)}}{{C\left( {w,T} \right)}} ,$$

where $C\left( {w,T} \right)$ is the cohesion measure from Eq. (11) for the whole network. According to Eq. (14) the highest curve has the highest betweenness. In this respect, Eq. (14) is more intuitive and the numerical values from Eq. (14) might be easier to compare with Eqs. (9, 10).

### Definition and algorithm for computing a community detection measure

The algorithm for community detection uses the influence measures $C_{s,t} ,\quad s,t = 1, \ldots ,N$ of Eq. (5) [equivalently in Eq. (7)]. The general method can be used also with other centrality measures presented in the literature. The idea in modelling community detection is based on the concept of node’s role in the network as a source and a target of influence. Both of these aspects have a role in community formation. Two sub-communities in a social network are detected by searching local maxima of Eq. (15):

15 $$P\left( {V,\bar{V}} \right) = \mathop \sum \limits_{i,t \in V} C_{s,t} + \mathop \sum \limits_{{i,t \in \bar{V}}} C_{s,t} ,$$

where $V$ and $\bar{V}$ is the split into two factions of the network of $N$ nodes with $N = N_{V} + N_{{\bar{V}}}$. We assume that these roles have equal importance in community formation. The community detection algorithm used in this study searches local maxima of Eq. (15) moving nodes, one at a time, that most increase the measure used for optimizing the division, between these factions.

Similarly, the classical Kernighan–Lin algorithm [28] is based on moving nodes between two factions of a network. However, Kernighan–Lin algorithm searches a community of pre-determined size and provides no sub-structures. In addition, the model of this paper calculates influence between all the nodes of the network as a function of time. Instead, the Kernighan–Lin algorithm is based on modularity maximization of the community and local topology of the network when determining which nodes to exchange between the two factions. Other community detection methods have been reviewed in [13], where strengths and weaknesses of modern methods are pointed out, and directions given to their use.

Typically, social networks with weak interactions between nodes, or social networks in their early development phases, have several local maxima with different compositions. These factions can overlap with each other. In many cases, unions and intersections of the divisions are also local maxima of Eq. (15) with some parameters of the model. If a union or intersection is not identified as a local maximum, these sets of nodes could still be considered as possible sub-groups of the network. In dynamic community building processes sets of nodes divided by different community boundaries may be left as outsiders. This is more probable if the measure of Eq. (15) has a low value or several divisions have almost equal numerical values.

Computing the community detection measure of Eq. (15) can be time consuming for large networks. This is a cost of considering influence spreading globally in the network. Several methods can be used to optimize the algorithm. First, limiting the computation to local nodes is an obvious alternative. Further, if a limited sub-set of the network is of interest, approximations can be computed by considering only the selected sub-set and some neighbouring nodes and structures around it.

The method for community detection consists of two independent main algorithms. The first algorithm is optimized for describing social influence spreading. The scalable version of the algorithm [11] allows loops in the process of influence spreading. The second algorithm uses results of the first algorithm. The input for the second algorithm is $N \times N$ matrix $C_{s,t}$ at time $T$, and control variables, if the analysis is limited to a specified portion of the network. This is relevant when very large social networks are investigated or a particular set of members of the social network are under investigation. Because the first algorithm is able to deal with large networks up to 100,000 nodes, matrix $C_{s,t}$ is usually computed for the entire network.

The default procedure is to compute $C_{s,t}$ for all the nodes of the network $\left( {s,t = 1, \ldots ,N} \right)$ and compute all the communities and sub-communities for the entire network (Step 1 below). From these results analysis and visualization can be focused on different sub-sets of the network (Step 2 below).

1. Compute the influence matrix $C_{s,t} ,\; s,t = 1, \ldots ,N$. Closeness and betweenness centrality measures are results of this step.
2. Compute the list of communities and sub-communities. Communities and their nested and overlapping structures are analysed.

Next, we present a basic version of the second algorithm for community detection.

1. Randomize values of vector $V$ of $N$ elements. Vector $N$ has elements of zeros and ones.
2. Use $V\left( n \right),\; n = 1, \ldots ,N$ as the initial state of the network. If $V\left( n \right)$ is one, node $n$ belongs to the first faction of the division, and if $V\left( n \right)$ is zero $n$ belongs to the complement of the faction.
3. Compute the community detection measure $P$ of Eq. (15). Denote the value of the initial state by $P_{0}$.
4. Starting from Node 1 move nodes from one faction to the other. Denote the value of $P$ by $P_{i}$ for *i*th move.
5. If the value of $P_{i}$ is higher than $P_{i - 1}$ move the node to the other faction, in other cases don’t move the node.
6. After all the nodes of the network have been computed, start from Step 4 again.
7. Repeat Step 6 while the value of $P$ is increasing else a local maximum has been found.
8. Repeat Steps 1–7 until a desired number of local maxima, or no new compositions, are found.
9. Analyse the list of detected communities. The list has the following information for every detected community: the value of $P$, sizes of the two communities, and the list of nodes for the detected communities. Nested and overlapping structures are discovered from the list of nodes.

A method to optimize the algorithm is to compute the list of communities in two phases. After detecting a desired number of communities with the basic algorithm, nested community structures are considered. In the second phase, in Step 2, the algorithm uses interceptions $C_{i} - C_{j}$ of detected communities $C_{i}$ and their detected sub-communities $C_{j}$, where $C_{j} \in C_{i}$. The intersections are often sub-communities or they are close to a composition of a sub-community. This makes computing times shorter because Steps 6 and 7 are less iterated.

Secondary effects between the two factions are included when computing the individual influence measure of Eq. (6). A variant of the model, would compute the two factions separately. This may better describe situations of the original social network splitting into two independent networks. The model presented in this paper is proposed for studying existing sub-communities and structures of a social network where interactions between sub-communities are continuous.

## Numerical results and discussion

Numerical results for the centrality measure of Eq. (9) and the betweenness measures of Eqs. (12–14) are compared with the results of [3]. The betweenness measures of Eqs. (12–14) are defined with the help of removing one node from a network. This ensures that the closeness centrality and betweenness measures are consistent with each other. The method of community detection measure is also based on the same formulation. Results of analysing community structures of four different networks are presented after the results for closeness centrality and betweenness centrality.

### Closeness centrality

**Fig. 3:**
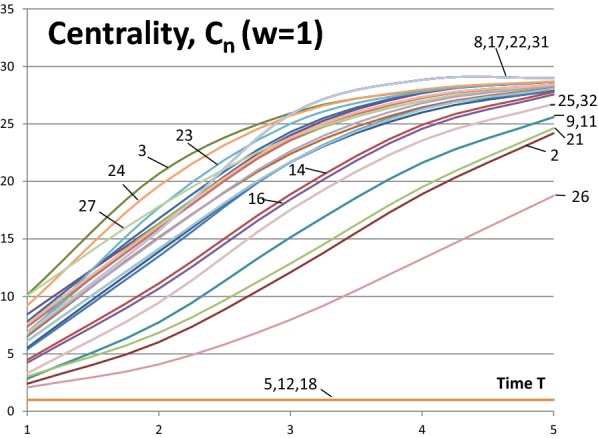
Centrality measures *C*_*n*_(*w*_*N*_ = 1.0) for the network of Fig. 1

First, we investigate the centrality measures $C_{n}$ of Eq. (9) and, later in the text, compare the results with Eq. (2) in [3]. Figure 3 shows the results of Eq. (9) as a function of time $T$. In this paper, the convention of value 1.0 is used for the node itself (in [3]) the contribution of the node itself is 0.0). This a matter of convention, the main results presented in this paper remain the same. It is a straight forward task to convert the numerical values between the two conventions. With full activity nodes ($w_{N} = 1.0$) all the centrality measures $C_{n}$ start from the value of one and approach the number of nodes in the network $N = 32$ with different rates depending on nodes’ positions in the network structure. Exceptions are the isolated nodes whose centrality value from Eq. (9) is constant 1.0.

**Fig. 4:**
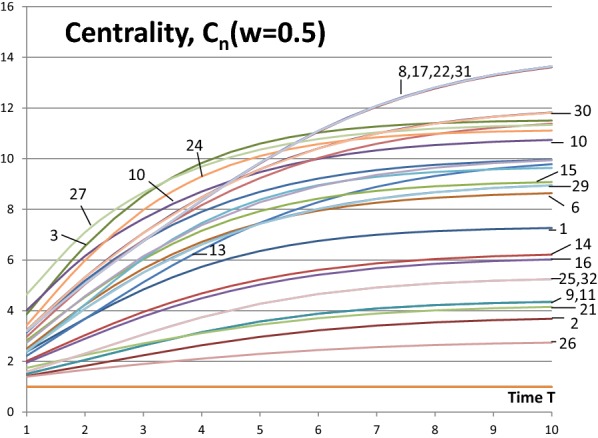
Centrality measures *C*_*n*_(*w*_*N*_ = 0.5) for the network of Fig. 1

Table 4 documents the values of centrality measure of Eq. (9) when the weighting factor is $w_{N} = 1.0$ and Table 5 documents the corresponding results when $w_{N} = 0.5$. In Table 4 centrality measures are listed when time $T = 1, \,3, \,5$ and in Table 5 when $T = 1, \,3,\, 10$. The results are shown in the order of node numbers to help comparison with the results in [3]. The right hand side of the tables shows the rankings of nodes. Tables 3 and 4 correspond to Figs. 3 and 4 in the sense that the results of the tables can be found in the figures at the time points $T$ given in the tables.

**Table 3:** Computing times of the community detection algorithm for searching 100 communities or sub-communities

Social media	Nodes	Links	Computing time
Facebook	4039	88,234	1 min
Enron e-mail	36,692	183,831	6 h

**Table 4:** Results *C*_*n*_(*w*_*N*_ = 1.0, *T*) from Eq. (9)

*n*	*w*_*N*_ = 1.0					
	*C*_*n*_*, T *= 1	*C*_*n*_*, T *= 3	*C*_*n*_*, T *= 5	Ranking, *T *= 1	Ranking*, T *= 3	Ranking*, T *= 5
1	0.173	0.677	0.872	3	3	8
2	0.075	0.378	0.756	27	8	17
3	0.317	0.810	0.896	24	17	22
4	0.238	0.744	0.888	10	22	31
5	0.031	0.031	0.031	7	31	24
6	0.204	0.700	0.880	4	24	23
7	0.244	0.759	0.895	30	23	10
8	0.214	0.807	0.906	20	7	3
9	0.089	0.473	0.800	15	10	7
10	0.264	0.751	0.896	8	4	4
11	0.089	0.473	0.800	17	30	30
12	0.031	0.031	0.031	22	27	20
13	0.168	0.678	0.869	31	20	15
14	0.140	0.590	0.868	28	15	27
15	0.215	0.736	0.886	23	28	28
16	0.132	0.576	0.861	6	6	6
17	0.214	0.807	0.906	19	19	19
18	0.031	0.031	0.031	29	29	29
19	0.191	0.678	0.877	1	13	1
20	0.230	0.737	0.888	13	1	13
21	0.095	0.402	0.769	14	14	14
22	0.214	0.807	0.906	16	16	16
23	0.208	0.782	0.896	25	25	25
24	0.287	0.804	0.897	32	32	32
25	0.103	0.548	0.835	21	9	9
26	0.065	0.249	0.586	9	11	11
27	0.315	0.744	0.886	11	21	21
28	0.211	0.707	0.884	2	2	2
29	0.191	0.678	0.877	26	26	26
30	0.238	0.744	0.888	5	5	5
31	0.214	0.807	0.906	12	12	12
32	0.103	0.548	0.835	18	18	18

When $w_{N} = 1.0$ and time $T = 1$ Nodes 3, 27 and 24 are the most central. Soon after, Node 24 is more central than Node 27. After time $T = 3$ Nodes {8, 17, 22, 31} are the most central nodes. These four nodes are in symmetrical positions in the network (indicated by the curly brackets) and have equal centrality values. These examples show that the most central nodes may change during the influence spreading process. This is a consequence of the network structure. At an early phase of the process Node 27 is more central because the spreading has just started and direct connections from the source node are emphasized. Node 27 has a high degree value of 8. Later Node 24 is more important in a central position between far away parts of the network even if its degree is only 4. The ranking of Node 27 is falling rapidly. Node 10 has similar changes but later at time $T = 5$ it is rising again because of the highly connected group of four Nodes {8, 17, 22, 31}.

We may be interested also about the least central nodes. Obviously, the isolate Nodes 5, 12 and 18 are the least central. Nodes 26 and 2 follow in this order. Next Nodes {9, 11} and 21 at time $T = 1$ and Nodes 21 and {9, 11} at times $T = 3, 5$ follow. Again network topology has its implications: Nodes 9 and 11 get benefits from their better connectivity at later development phases of the influence spreading. Curly brackets indicate that Nodes 9 and 11 are at symmetrical positions in the network structure.

Next, we examine whether less active nodes in the network behave in a similar way. In Fig. 4 the values of the centrality measure of Eq. (9) with node activities $w_{N} = 0.5$ are shown. The same results and rankings at times $T = 1, \,3,\, 10$ are listed in Table 5. The role of the highly connected group of Nodes {8, 17, 22, 31} is even more emphasized at a later time $T > 6$ but their role is less important at the beginning of the influence spreading process. We make a conclusion that peripheral interconnected nodes’ centrality at high values of time (near equilibrium state) is relatively higher for low activity networks than for high activity networks.

**Table 5:** Results *C*_*n*_(*w*_*N*_  = 0.5, *T*) from Eq. (9)

*n*	*w*_*N*_ = 0.5					
	*C*_*n*_, *T* = 1	*C*_*n*_, *T* = 3	*C*_*n*_, *T* = 10	Ranking, T = 1	Ranking, T = 3	Ranking, T = 10
1	0.074	0.151	0.227	27	27	8
2	0.045	0.070	0.115	10	3	17
3	0.121	0.267	0.359	3	24	22
4	0.101	0.221	0.369	24	10	31
5	0.031	0.031	0.031	4	4	4
6	0.078	0.177	0.270	30	30	30
7	0.098	0.211	0.311	8	20	3
8	0.100	0.211	0.426	17	7	20
9	0.047	0.082	0.136	22	8	27
10	0.125	0.238	0.336	31	17	24
11	0.047	0.082	0.136	7	22	10
12	0.031	0.031	0.031	20	31	7
13	0.069	0.160	0.306	15	28	28
14	0.063	0.124	0.194	28	23	13
15	0.088	0.188	0.284	6	15	23
16	0.061	0.118	0.188	19	6	15
17	0.100	0.211	0.426	29	19	19
18	0.031	0.031	0.031	1	29	29
19	0.076	0.172	0.280	23	13	6
20	0.093	0.212	0.356	13	1	1
21	0.054	0.085	0.130	14	14	14
22	0.100	0.211	0.426	16	16	16
23	0.074	0.190	0.301	21	25	25
24	0.107	0.249	0.347	25	32	32
25	0.050	0.096	0.164	32	21	9
26	0.044	0.059	0.086	9	9	11
27	0.145	0.271	0.354	11	11	21
28	0.085	0.192	0.311	2	2	2
29	0.076	0.172	0.279	26	26	26
30	0.101	0.221	0.369	5	5	5
31	0.100	0.211	0.426	12	12	12
32	0.050	0.096	0.096	18	18	18

Table 6 reiterates some results from Table 1 in [3] in the same format as in Tables 3 and 4 of this paper. Columns show the results of Eq. (2) when $\delta = 5,\, 1, \,0.5$. Nodes at a longer geodesic distance become less important for high values of $\delta$ [3]. This is the reason for presenting the results in this order to help comparing with the results of this paper when time $T$ increases. Note that the numerical values cannot be compared directly because of the different definitions of measures in Eqs. (2) and (9).

**Table 6:** Results of generalized closeness centrality with different from Table 1 in [3]

*n*	*δ* = 5	*δ* = 1	*δ* = 0.5	Ranking, *δ* = 5	Ranking, *δ* = 1	Ranking, *δ* = 0.5
1	0.103	0.354	0.548	27	27	3
2	0.034	0.227	0.439	10	3	27
3	0.175	0.469	0.635	3	24	24
4	0.135	0.355	0.539	24	10	10
5	0.000	0.000	0.000	7	7	7
6	0.074	0.339	0.534	4	15	15
7	0.138	0.396	0.578	15	23	23
8	0.132	0.327	0.516	30	4	1
9	0.035	0.257	0.469	8	30	4
10	0.199	0.415	0.586	17	1	30
11	0.035	0.257	0.469	22	20	6
12	0.000	0.000	0.000	31	6	20
13	0.067	0.267	0.469	20	28	28
14	0.07	0.305	0.506	1	8	8
15	0.135	0.376	0.561	28	17	17
16	0.069	0.297	0.500	6	22	22
17	0.132	0.327	0.516	19	31	31
18	0.000	0.000	0.000	23	19	19
19	0.072	0.317	0.516	29	29	29
20	0.104	0.339	0.530	14	14	14
21	0.066	0.253	0.456	16	16	16
22	0.132	0.327	0.516	13	13	25
23	0.072	0.359	0.558	21	25	32
24	0.140	0.430	0.609	25	32	13
25	0.036	0.265	0.476	32	9	9
26	0.034	0.199	0.408	9	11	11
27	0.264	0.470	0.623	11	21	21
28	0.103	0.334	0.526	2	2	2
29	0.072	0.317	0.516	26	26	26
30	0.135	0.355	0.539	5	5	5
31	0.132	0.327	0.516	12	12	12
32	0.036	0.265	0.476	18	18	18

To compare the results we try to find corresponding columns from the tables. This is not exactly unambiguous because the functional relationship between $\delta$ in Eq. (2) and $T$ in Eq. (9) is not known. Probably, no exact functional form exists because the structure of a network can produce complex effects on the functional form. We provide an example how the results can be compared. The results are remarkably similar when the rankings of the most central nodes are compared. However, there are some distinctive differences.

Because development phase of the social network is not known, it is not possible to determine the time value $T$. We could examine all the possible time values $T$ and compare with the results from Eq. (2) with all the possible values of $\delta$. On the other hand, Eq. (2) is not describing dynamic development of the spreading process. The model of this paper is dynamic and the model of [3] is static. As a consequence, full analysis is not necessary. Instead we give an example that illuminates some similarities and differences of the results. For comparison, we choose one value of $\delta$. Then we search from Tables 3 and 4 time values (columns) that provide roughly the same rankings of the most central nodes and conclude that these time values correspond to the results from Eq. (2) with the value of $\delta$.

The first line in Table 7 shows the ranking results from Eq. (2) [3] with the parameter value of $\delta = 0.5$. Results from Eq. (9) of this paper are shown on the second line with the parameter values of $w_{N} = 1.0$ and $T = 1$ and on the third line with $w_{N} = 0.5$ and $T = 3$. These two lines approximately correspond to the first line. It is noticeable that in an active network with $w_{N} = 1.0$ a shorter development time $T$, compared with a less active network with $w_{N} = 0.5$, is required to achieve approximately the same rankings of central nodes in the network. The group of highly interconnected Nodes {8, 17, 22, 31} is peripheral in the network structure. These nodes are underestimated in [3] when compared with results of Eq. (9) in Table 7.

**Table 7:** An example summarized from Tables 3, 4 and 5

Equation (2), *δ *= 0.5	3	27	24	10	7	15	23	1	4	30	6	20	28	{8, 17, 22, 31}
Equation (9), *w*_*N*_ = 1.0,* T* = 1	3	27	24	10	7	4	30	20	15	{8, 17, 22, 31}	28	23	6	19
Equation (9), *w*_*N*_ = 0.5, *T * = 3	27	3	24	10	4	30	20	7	{8, 17, 22, 31}	28	23	15	6	19

Also the parameter value of $\delta$ and the time value $T$ are related: high values of $\delta$ correspond low values of $T$. This can be seen when comparing column $T = 1$ in Table 5 with column $\delta = 5$ in Table 6. Both have the same most influential Nodes {27, 10, 3, 24}. The same comment as above concerning Nodes {8, 17, 22, 31} holds also for $\delta = 5.$

### Betweenness centrality

Betweenness measures node’s role as a broker between others. In Eq. (12), we have presented a new betweenness measure with the help of removing one node from the network. An alternative presentation in Eq. (14) is normalized by the value of Eq. (11) describing network structure where all the nodes are present.

**Fig. 5:**
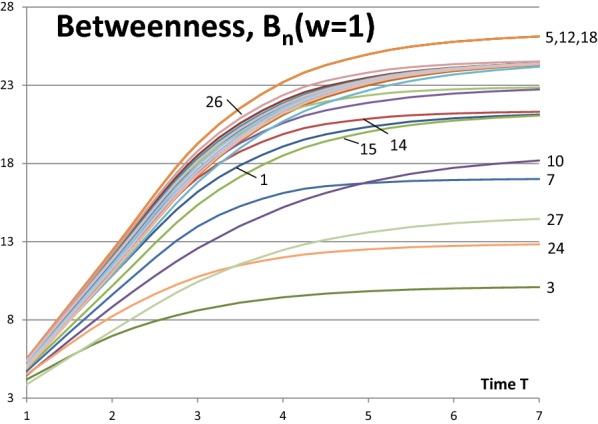
Betweenness measures *B*_*n*_(*w*_*N*_ = 1.0) of Eq. (12) for the network of Fig. 1

**Fig. 6:**
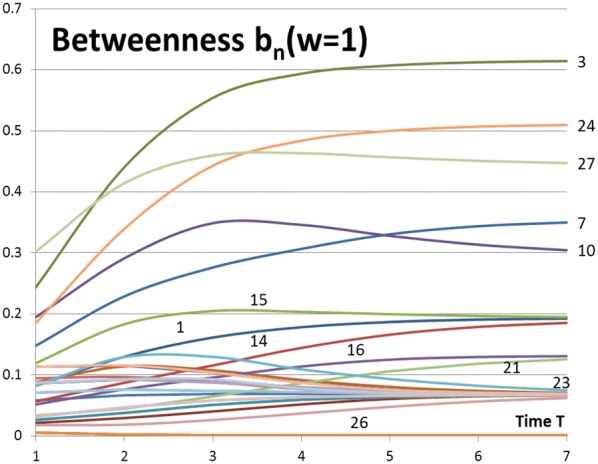
Betweenness measures *b*_*n*_(*w*_*N*_ = 1.0) of Eq. (14) for the network of Fig. 1

Results of Eqs. (12) and (14) for the network of Fig. 1 are shown in Figs. 5 and 6. Network activity is $w_{N} = 1.0$ in both figures. Notice that the lowest (highest) curves in Fig. 5 (Fig. 6) represent the highest betweenness of nodes. The rankings of betweenness values are the same in both approaches. As can be seen from Figs. 3 and 5 closeness centrality and betweenness centrality describe different characteristics of the network. The most central node is not always the best broker of influence in the network. But in many cases a node can have both of these characteristics at the same time.

**Fig. 7:**
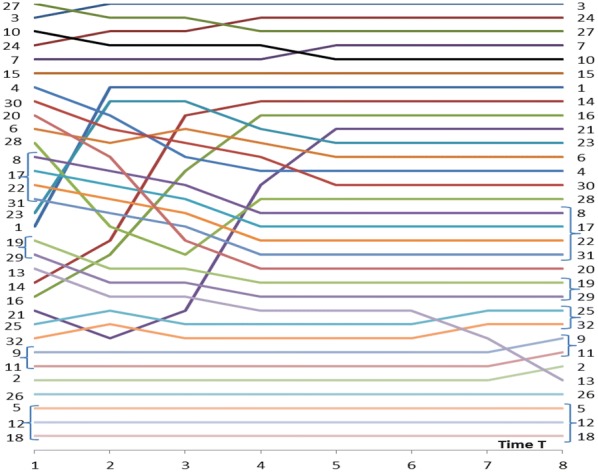
Rankings of nodes according to betweenness with *w*_*N*_ = 1.0 for the network of Fig. 1

**Fig. 8:**
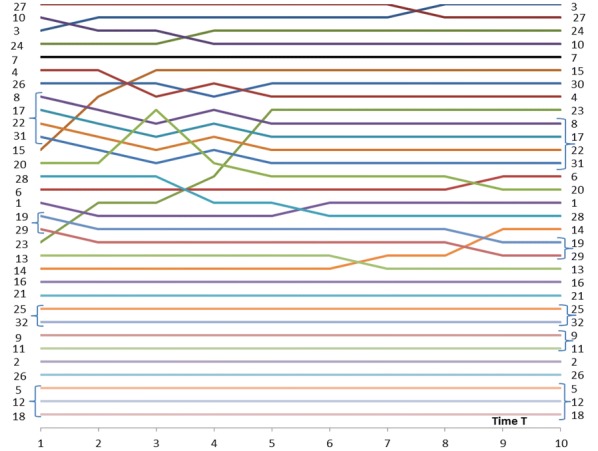
Rankings of nodes according to betweenness with *w*_*N*_ = 0.5 for the network of Fig. 1

Rankings of betweenness values are shown in Figs. 7 and 8 for the activity values of $w_{N} = 1.0$ and $w_{N} = 0.5$ correspondingly. Figures 5, 6 and 7 show the same information of betweenness with $w_{N} = 1.0$ in different formats. From Fig. 1 we can see that Nodes 3, 24 and 27 are nodes having a good location between others. Nodes 10 and 27 have more important roles as brokers at the beginning of the spreading process. They are in a good position as brokers between highly connected peripheral nodes and rest of the network. Figures 7 and 8 highlight the complex behaviour influence spreading processes as a function of time. Betweenness rankings can move in turn up and down depending on the development phase of the process.

## Results for community detection

### Structure of the territory network of the game of risk

The game of Risk has also been used in the literature [37] as one of test networks for community discovery algorithms. The network is neither a human nor an animal social network that is why real life interpretation for model parameters may not be valid. This network is an example of analysing network structures of general networks, not just social networks and communities. On the other hand, this artificial network turns out to be the simplest of our four example networks. Investigating the social network of 32 Dutch students is analysed after presenting basic ideas with the help of the Game of Risk network structures.

The board game is played on a political map consistently of six continents which further divide into 43 territories. The territories are connected by boundaries and waterways. The goal of the game is to conquer as much land as possible.

In the model, we presume some reasonable parameter values that provide a number of divisions of the territories network structure. The value of time is $T = 1.0$ and the parameter values are $\lambda = 0.5,w_{N} = w_{L} = 1.0,{L_ \text{max} } = 4$. This choice of parameters provides us 17 different divisions of the 42 territories listed in Table 8. The numbers of territories in the divisions are shown in the column indicated by ‘#’. The values of the measure of Eq. (15) give the rankings (first column) of the divisions. The results of Table 8 are interpreted in the order of the ranking values.

**Table 8:** The 17 different divisions of the 42 territories of Risk game detected as local maxima of Eq. (15)

				North America									Europe							Asia											
*R*	#	#	Equation (15)	Nodes																											
*1*	38	4	128.0	1	2	3	4	5	6	7	8	9	10	11	12	13	14	15	16	17	18	19	20	21	22	23	24	25	26	27	28
2	4	38	124.4																												
3	33	9	122.8										10	11	12	13	14	15	16	17	18	19	20	21	22	23	24	25	26	27	28
4	34	8	122.3	1	2	3	4	5	6	7	8	9	10	11	12	13	14	15	16	17	18	19	20	21	22	23	24	25	26	27	28
5	13	29	121.1	1	2	3	4	5	6	7	8	9																			
6	29	13	120.6										10	11	12	13	14	15	16	17	18	19	20	21	22	23	24	25	26	27	28
7	25	17	119.0										10	11	12	13	14	15	16	17	18	19	20	21	22	23	24	25	26	27	28
8	6	36	118.2																		18	19	20	21			24	25			
9	10	32	116.3																		18	19	20	21			24	25			
10	15	27	116.1	1	2	3	4	5	6	7	8	9									18	19	20	21			24	25			
11	19	23	114.2	1	2	3	4	5	6	7	8	9									18	19	20	21			24	25			
12	15	27	114.1																	17	18	19	20	21	22	23	24	25		27	28
13	23	19	112.5	1	2	3	4	5	6	7	8	9									18	19	20	21			24	25			
14	32	10	112.4	1	2	3	4	5	6	7	8	9	10	11	12	13	14	15	16	17					22	23			26	27	28
15	18	24	112.2										10	11	12	13	14	15	16										26		
16	11	31	112.1																	17	18	19	20	21	22	23	24	25		27	28
17	14	28	110.6										10	11	12	13	14	15	16										26		

				South America				Africa						Australia			
*R*	#	#	Equation (15)	Nodes													
1	38	4	128.0	29	30	31	32	33	34	35	36	37	38				
2	4	38	124.4	29	30	31	32										
3	33	9	122.8	29	30	31	32	33	34	35	36	37	38	39	40	41	42
4	34	8	122.3					33	34	35	36	37	38				
5	13	29	121.1	29	30	31	32										
6	29	13	120.6	29	30	31	32	33	34	35	36	37	38				
7	25	17	119.0					33	34	35	36	37	38				
8	6	36	118.2														
9	10	32	116.3											39	40	41	42
10	15	27	116.1														
11	19	23	114.2											39	40	41	42
12	15	27	114.1											39	40	41	42
13	23	19	112.5	29	30	31	32							39	40	41	42
14	32	10	112.4					33	34	35	36	37	38	39	40	41	42
15	18	24	112.2	29	30	31	32	33	34	35	36	37	38				
16	11	31	112.1														
17	14	28	110.6					33	34	35	36	37	38				

The values of the measure in Eq. (15) are shown in the table with the number nodes in the two factions of the network

The title line in the table shows the continents where the 42 territories are located (this information is not used as input in the model)

The first five lines in Table 8 show clearly continents Australia, South America and North America. On lines 1–7 Europe, Africa and Asia are joined together. Not until on line 12 Asia without territory 26, is identified as a division. In addition sub-communities of five {17, 22, 23, 27, 28} and six {18, 19, 20, 21, 24, 25} territories are identified within Asia.

The algorithm, with the parameters used, does not discover Europe and Africa as individual divisions. Node 26 is incorrectly identified to the combined coalition of Europe and Africa. Classifications in the literature have been referenced in [41] where three out of five algorithms, FastQ, LPA and PPC, also misclassify Node 26. Also three algorithms LPA, Infohiermap and PPC extract the same sub-communities of five and six nodes in Asia. Two algorithms, Infohiermap and the active semi-supervised algorithm of [41], identify Europe and Africa and their territories correctly.

**Fig. 9:**
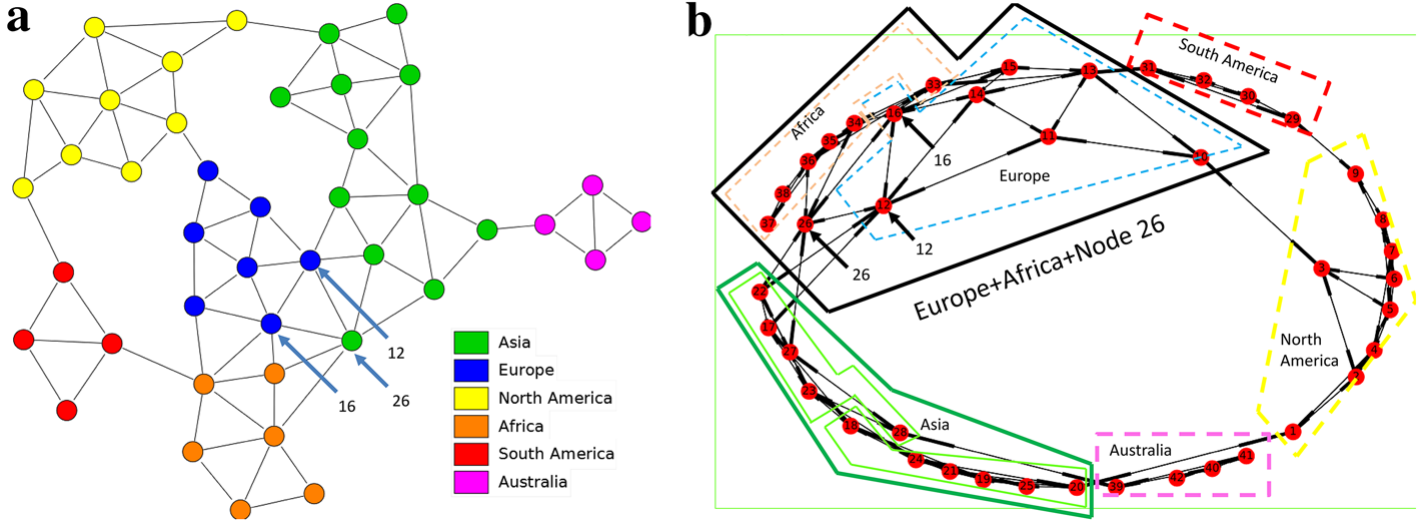
**a** The network of 42 territories in Risk game where colours indicating the six continents (Wikipedia Commons). **b** The same network automatically generated by a library of Python software package together with the five divisions marked on the network as discovered by the algorithm of this paper. Europe and Africa are identified as one faction by the algorithm

When investigating the territory network we observe that Nodes 16 and 26 (see Fig. 9a, b) are critical territories between Europe and Africa. They produce strong interrelations between these continents. Using lower values of the node weighting factors does not change the results as Europe and Africa still appear in the same group in different divisions.

We summarize the results of analysing the artificial Risk game network with the proposed model. Characteristics of human social networks are not assumed to be valid but we can compare the results with other algorithms in the literature. The results are similar, with an exception of two factions of the network identified as one group. Few other algorithms detect the correct nodes of the two communities although they detect the communities themselves [41]. Some of the algorithms in the literature use supervised or assisted methods which can lead to more accurate results.

In analysing Risk game network we use a tabular form of representing different community structures in the network. This is an illustrative and useful way of detecting communities and sub-communities in a social network. The role of numerical values of the community detection measure of Eq. (15) is highlighted by examining lines of the table in the order of numerical values of the measure.

### Community structures of the 32 Dutch students’ social network

As the second case for analysing community structures, we use the longitudinal friendship network among 32 Dutch students on the fourth wave of the collected data [36]. Two students are considered to be friends if either or both of them named the other as a friend. A graphical representation of the friendship network is shown in Fig. 1.

The social network is analysed with two different model parameters $w_{N} = 0.5$ and $w_{N} = 1.0$ describing strength of the friendship relations. In both cases the parameter values $\lambda = 0.5, T = 1.0, w_{L} = 1.0$ and ${L_ \text{max} } = 6$ are used. These parameter values are used for all connections in the network.

**Fig. 10:**
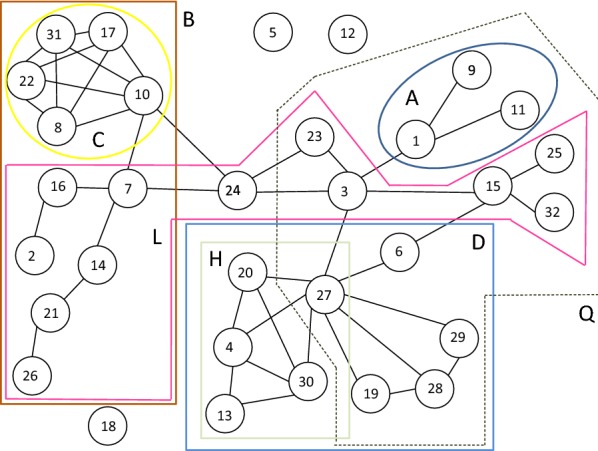
Representative sub-communities detected among the 32 Dutch students’ social network

Sub-communities detected among the 32 Dutch students’ social network are presented in Table 9 and in Fig. 10 for the two values of node weighting factors. The results for $w_{N} = 0.5$ has 14 different divisions and $w_{N} = 1.0$ has 6 divisions of the network. The first two columns show the number of nodes in the two factions, the third column shows the label of the division and the fourth column shows the numerical value of the community detection measure of Eq. (15). The results are presented in descending order of these values. The nodes belonging to one of the two sub-divisions is indicated in the table, the rest of the nodes belong to the second sub-division. Actually only 29 nodes out of the 32 nodes are connected. Nodes 5, 12 and 18 have no connections and they are not included in the sizes of the sub-divisions in Table 9.

**Table 9:**
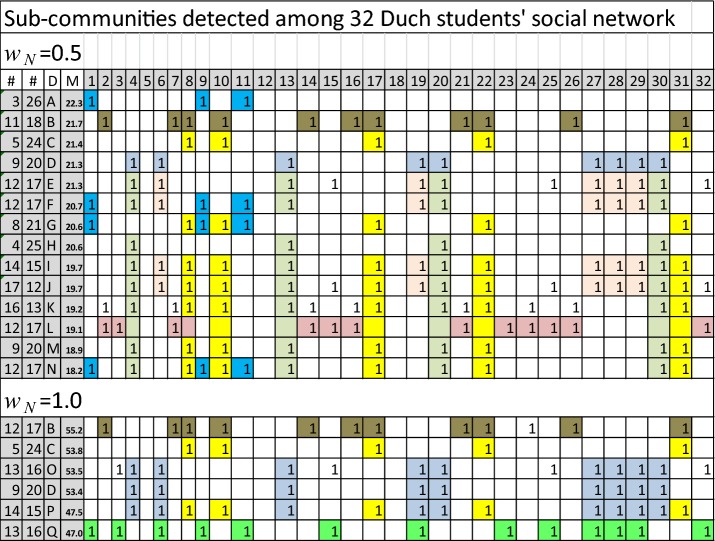
Different divisions into two factions of the social network of Fig. 1

Model parameters are *W*_*N*_ = 0.5 and *W*_*N*_ = 1.0 with *T* = 1.0, *λ* = 0.5, *W*_*L*_ = 1.0, *L*_max_ = 6

For weaker connections of $w_{N} = 0.5$ (or for low values of spreading time $T$) the highest value of the local maximum value is $M = 22.3$ for the two factions {1, 9, 11} and {2, 3,…, 8, 10, 12,…32}. This division is not a local maximum for stronger connections of $w_{N} = 1.0$ at the same time $T = 1.0$. These kinds of weakly connected small sub-groups can exist at an early development phase of friendship relations.

A larger division *B*, on the left side of Fig. 1, into 11 and 18 nodes can be discovered for both weak $w_{N} = 0.5$ and strong $w_{N} = 1.0$ connections. This is also the case for the tightly connected sub-group {8, 10, 17, 22, 31} of division *C*. The value of the community detection measure for division *D* is almost as high as for *C*, even though division *O* with the four additional nodes $\left( {O = D \cup \{ 3,15,25,32} \right)$ has a slightly higher value for strongly connected $w_{N} = 1.0$ network. Almost similar to division *O* division *E* $\left( {E = D \cup \{ 15,25,32} \right)$ can be found for weak connections but not for strong connections.

Node 3 is a gateway node with high betweenness values in Figs. 5, 6 and 8. Node 3 is a member of sub-groups in exceptional sub-groups of divisions *L* and *Q*. The sub-group of nodes {2, 3, 7, 14, 15, 16, 21, 23, 24, 25, 26, 32} in division *L* rules out three separate factions *A, C,* and *D*. Other examples of unconnected factions can be found in Table 9 in divisions *F, G, I, J, K, M, N,* and *P*. In these cases a strongly connected sub-group disconnects the second faction of the division. In this way more than two separate sub-groups can build up as a result of dynamic behaviour of social networks.

In a typical situation sub-groups are nested, for example, $A \subset F,G,N,Q$, $C \subset G,I,J,K,M,N,P$ and $D \subset E,F,I,J,O,P.$ Often, sub-groups are unions, for example, $F = A \cup D$, $G = A \cup C$, and $N = A \cup C \cup H$. In many cases, differences of combined sub-groups are stand-alone sub-groups. However, for example, {15, 25, 32} and {2, 7, 14, 16, 21, 26} are not separate sub-groups in any divisions. This can be sensitive to model parameters.

### Community structures of a social network of dolphins

The third dataset we have selected to test the proposed community structure algorithm is the data of dolphin association collected for a research programme [38] of a community of 62 bottlenose dolphins over a period of 7 years. The network describing interactions of dolphins represents one of the real-world networks for which the community structure is already known. The social network has been analysed in [42] with a method published in [43, 44]. Two communities and four sub-communities were detected in the dolphin network. A temporary disappearance of the dolphin denoted by SN100 led to the fission of the dolphin community into two factions.

The animal social network is found to be similar to a human social network in some respects but different in some others such as the level of assortative mixing by degree within the population. Assortative mixing by age but not by vertex is observed in the dolphin social network [42]. Assortative mixing is a bias in favour of connections between network nodes with similar characteristics in complex networks [45]. In the model of this paper this may favour a lower value of maximal path length for the dolphin social network than for human social networks.

**Fig. 11:**
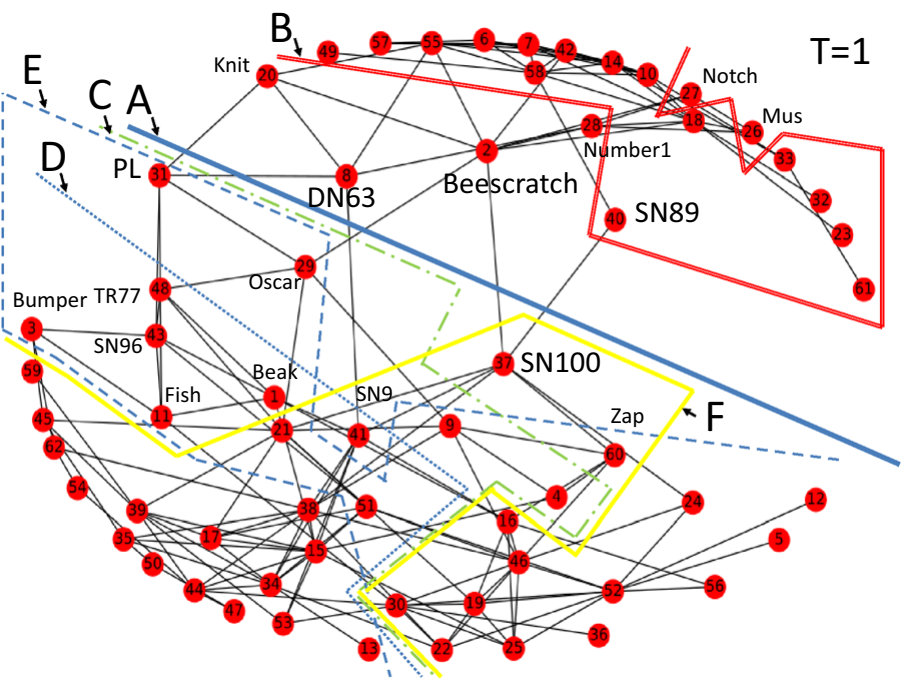
Communities discovered from the dolphins’ social network [42]. Time value  *T* = 1.0 and node weighting factors *W*_*N*_ = 0.5 are used with model parameter values  *λ* = 0.5, *L*_max_ = 4

In Table 10 factions of nodes producing six local maxima in the values of the measure of Eq. (15) are listed. Boundaries of these divisions are shown in Fig. 11. The highest value is for division A which is the split observed in real life, with one exception of Node SN89, after dolphin SN100 temporarily disappeared from the original dolphin community. Out of the five additional less optimal divisions almost as good division B separates a smaller group of more peripheral 15 nodes. This indicates a mediating role of the six dolphins Beescratch, DN63, Knit, Mus, Notch and Number1.

**Table 10:** Optimal divisions of the dolphin social network

Division	Network	A	B	C	D	E	F
Factions	0 + 62	21 + 41	15 + 47	35 + 27	39 + 23	38 + 24	22 + 40
Eq. (15)	122.2	115.9	114.0	94.9	93.9	92.6	92.5

Division *C* is an analogous division on the other side of the main spit boundary of dolphin SN100 and a group of 12 closely interconnected dolphins on the right lower part of Fig. 11. These 12 nodes are exactly the nodes identified by [42] as a sub-community in the network. There are also three additional more divisions *D, E,* and *F* indicated in Fig. 11.

We have computed also the results with Node SN100 removed from the original network. The results are almost similar. The divisions *A* and *B* are the same as in Fig. 11. Division *C* has one Node Zap moved side. The fourth division of 16 nodes is in the lower left corner of Fig. 11 with dolphin SN9. The fifth and sixth divisions are exactly divisions *D* and *E* in Fig. 11.

Betweenness of nodes in the dolphin social network have been studied in [46]. Values of Eq. (12) have been calculated for two different parameter values describing low and high cohesion of the network. The results are very different for these two cases. At early phases of influence spreading different nodes have the highest betweenness, when compared with later phases of the process, because later more nodes have already been affected. At early phases local characteristics and neighbouring nodes are controlling the spreading processes. Node degree is describing centrality in these situations.

In fact, the highest node degrees of the dolphin social network are for dolphins Grin, SN4 and Topless with 12, 11 and 11 node degree values correspondingly. In the low cohesion network, the nine nodes with the highest values of betweenness measure are {Grin, SN4, Topless, Scrabs, SN9, Kringel, Patchpac, Trigger, TR99}.

In the high cohesion network, the eleven nodes with the highest values of betweenness measure are {SN100, Beescratch, SN9, Trigger, SN9, Trigger, SN4, Jet, Scrabs, Stripes, Kringel}. These results are in agreement with the results in [42] identified using the betweenness-based algorithm of [43].

Low cohesion exists at an early phase of influence spreading or when nodes’ activities are low, i.e. low node weighting factors. A result of this is that corresponding pairs of time and weighting factor values can be found such that they provide comparable results. In [46] this has been demonstrated in cases of low values of time with high values of weighting factors, and high values of time with low values of weighting factors. Almost identical results are obtained for $T = 1.0,w_{N} = 1.0$ and $T = 4.5,w_{N} = 0.5$.

According to the research article [42] the dolphin community has existed quite a long time. On the other hand, the positive assortative mixing by degree was not observed in the study, which is often observed in human social networks. However, a clear statistically significant assortative mixing by sex among the dolphin population has been observed, although the mixing is not as strong as some types of mixing in human societies [42, 45].

We conclude from the results of [42], because of the lack of positive assortative mixing by degree, that relatively low value of $T = 1.0$ is appropriate. The value of $w_{N} = 0.5$ for node weighting factors are used in Fig. 11. We have made experiments with higher values of weighting factors and higher values of time. In latter development phases of influence spreading local maxima of community detection in Eq. (15) are levelled and fewer sub-communities are discovered. At time $T = 1.0$ with $w_{N} = 1.0$ only one division is detected which is exactly the same Division *A* in Fig. 11. Using the method for low and high cohesion circumstances can be regarded as a method to examine a network with diverse resolutions.

We summarize the results of analysing the dolphin social network. The split of the dolphin population into two factions after a temporal disappearance of dolphin SN100 is predicted by the model with the exception of one dolphin SN89. In the literature, sub-communities have been identified using the betweenness-based algorithm in [43]. The proposed model of this study does not predict the same sub-communities but they can be identified by the help of investigating boundaries of different divisions predicted by the model.

There is a question whether the same model parameters are appropriate for dolphin and human social networks. We conclude from the research published in [42] that low values of time $T$ or node weighting factors $w_{N}$ might be more appropriate for dolphin social networks. The same reasoning applies to maximum path length $L$ used in the model. In the proposed model, the consistent procedure is to use the same parameters for the community detection algorithm and for closeness and betweenness centrality measures.

### Community structures of a Facebook social network

In this section we show results for a social network of Facebook. This dataset consists of ‘circles’ (or lists of friends). The network data have 4039 nodes and 88,234 links between nodes. The data also have nested and overlapping communities. Here, our main focus is on presenting the features of our model and methods for larger social networks. Because of the detailed modelling, where all the nodes are considered, when influence between nodes are calculated, complex phenomena appear which may not be present in very small social networks. We also provide strategies on how to optimize the community detection calculations to minimize computer running times. The Facebook social network is the same as used in [11].

The analysis is conducted with the entire social network data, with loops allowed (except self-loops), maximum path lengths $L_{ \text{max} } = 6$, node weighting factors $w_{N} = 0.1$, and link weighting factors $w_{L} = 1.0$. The community detection measure of Eq. (15) is computed along the paths determined by the 88,234 links between nodes. As a result of the influence spreading process, all the nodes inside the maximum path length $L_{ \text{max} }$ in a connected graph are influenced by a node and the corresponding elements of the $N \times N$ matrix $C_{s,t}$ have positive values. The full analysis of the network considers all these elements.

All the information for detecting communities and their relations in a network consisting of $N$ nodes is included in one $N \times N$ matrix, which has influence measures of Eq. (6) from $N$ nodes to all the other nodes in the network. Because diagonal elements of the matrix have no effect on the community structure, we set the diagonal elements to zero. We show selected results of detailed structures of the network while the calculations have been conducted using the whole network data. Therefore, the method is global, not local, in this respect. This means that all the interactions have influenced the results, centrality and betweenness measures and community structures.

We have detected 551 sub-structures in the network. Most of these are nested structures inside communities. In large networks, many levels of nested sub-communities can exist. The algorithm provides list of nodes included in the 551 communities and the corresponding values of the community detection measure of Eq. (15). We order the communities by the values of the measure and use the ranking of a community as a unique identifier (in this section only smaller factions of divisions are studied). The analysis should start from important communities, their internal structures and relationships with other communities.

**Fig. 12:**
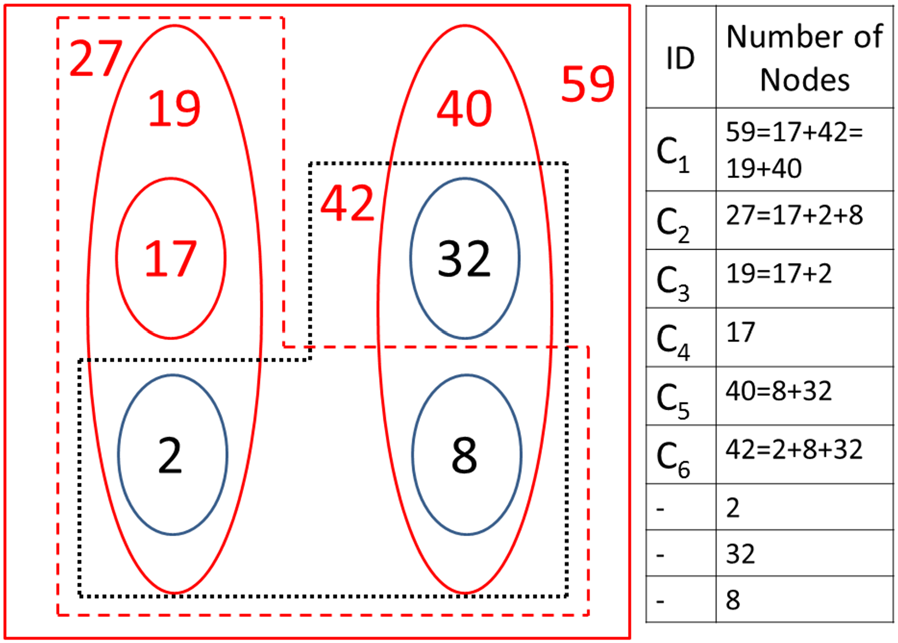
The five sub-communities *C*_2_,…, *C*_6_ detected in community *C*_1_. Numbers of nodes and community identifiers (ID) are shown. The sets of 2, 8 and 32 nodes are not detected as sub-communities with the used model parameters. These sets of nodes are intersections of the detected sub-communities

**Fig. 13:**
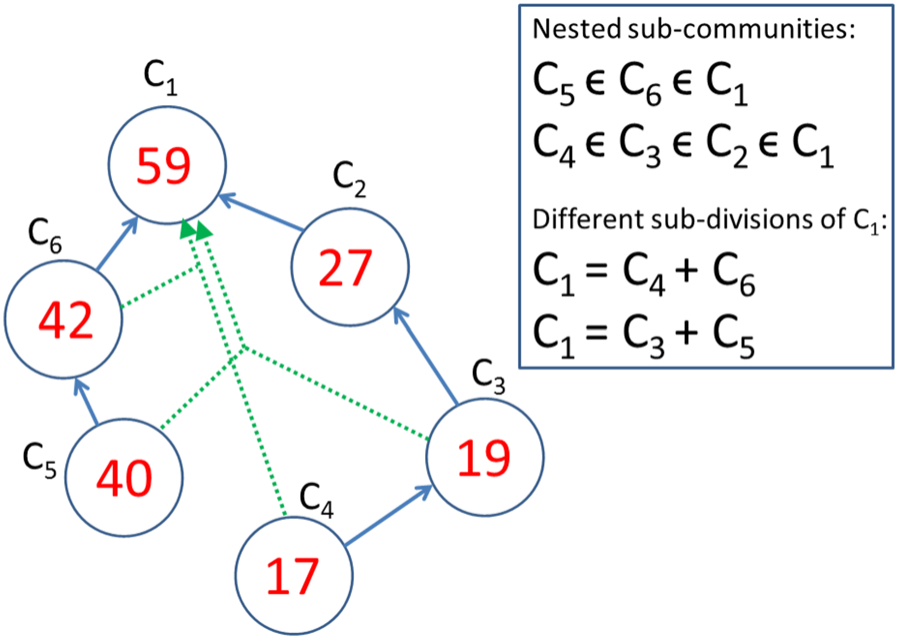
Nested sub-communities and different divisions of community *C*_1_ are shown with solid and dotted lines correspondingly

The smaller faction of the division with the highest value of the community detection measure of Eq. (15) consists of the 59 last nodes {3981–4039} of the network data. The value of the un-normalized measure of Eq. (15) is 442,159. This community has also complex sub-structures. Figures 12 and 13 show the overlapping and nested structures among the 59 nodes. Community $C_{1}$ and its sub-communities $C_{2} , \ldots ,C_{6}$ have a significant influence on community structures outside $C_{1}$, and in many cases, one or more of $C_{1} , \ldots ,C_{6}$ are included as sub-sets in these communities.

Nested sub-communities are indicated in Fig. 13 as $C_{5} \in C_{6} \in C_{1}$ and $C_{4} \in C_{3} \in C_{2} \in C_{1}$. Two different divisions of the 59 nodes are shown with dotted lines in Fig. 13 as $C_{1} = C_{4} + C_{6}$ and $C_{1} = C_{3} + C_{5}$. As can be seen in Fig. 12, three genuine overlapping cases exist: $C_{6} - C_{5} = S_{2}$, $C_{2} - C_{3} = S_{8}$, and $C_{5} - C_{2} = S_{32}$. We denote these intersecting sets of nodes by $S_{2}$, $S_{8}$, and $S_{32}$ because they have not been detected as sub-communities [local maxima of Eq. (15)] and have no community identifier in Fig. 12. However, together the three sets form sub-community $C_{6}$, denoted by $C_{6} = S_{2} + S_{8} + S_{32}$. Again, these sets of nodes may appear as sub-communities with lower parameter values. In fact, intersections of detected communities are candidates for new sub-communities.

**Fig. 14:**
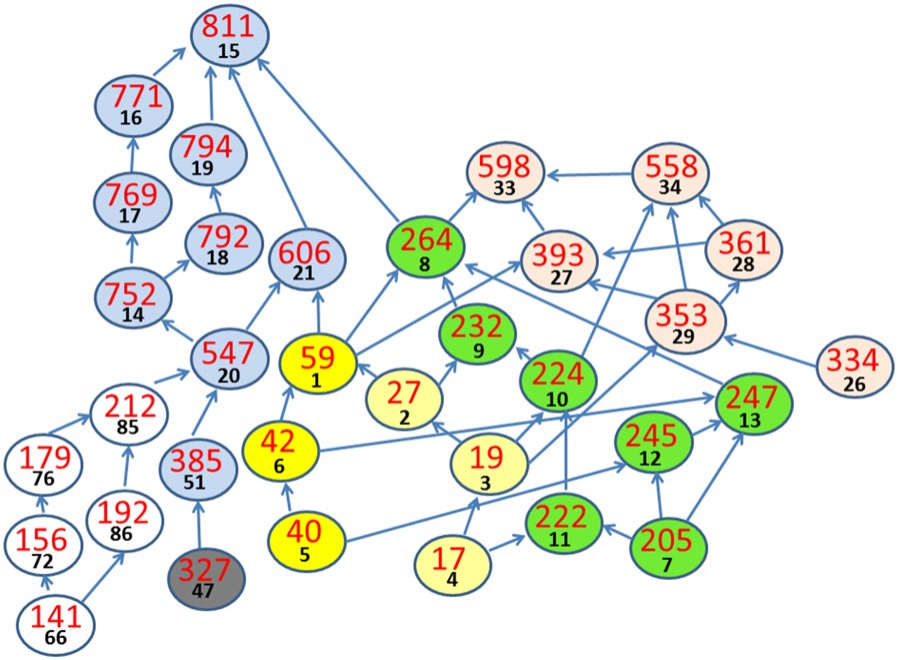
A sample of community structures in the Facebook social network. Selected sub-communities are shown with information about community sizes and community identifiers (rankings of communities). Arrows indicate nested structures of sub-communities. Analysis has been conducted with the entire network data of the 4039 nodes. Colouring is explained in Fig. 15

Figure 14 shows a sample of the analysis of the Facebook social network around the 59 nodes. Sub-communities are marked by their sizes and identifiers [rankings calculated from Eq. (15)]. In the calculations all the 4039 nodes have been considered. This means that the sub-communities and their compositions are probably different if only the nodes in sub-communities shown in Fig. 14 are considered. Figure 14 shows the nested structures of the sub-communities as in Fig. 13. Compositions of the sub-communities (dotted lines in Fig. 13) are not shown in Fig. 14.

In many cases a community has nested structures composed of sub-communities lower in hierarchy like the two examples in Fig. 13. This is a consequence of the fact that influence spreading is considered globally or at least inside the path length $L_{ \text{max} }$, if it is not set to infinity. Also, when a nested sub-community is detected inside a community, the other faction $S$ may not be detected as a sub-community. An example is shown in Fig. 12 where $C_{3} = S_{2} + C_{4}$. This does not exclude the possibility that $S$ is a constituent of a sub-community on higher levels. An example in Fig. 14 is the sub-community $C_{8}$ of 264 nodes composed of sub-community $C_{9}$ and a set of 32 nodes $S_{32}$ $\left( {C_{8} = S_{32} + C_{9} } \right)$. This is possible because community $C_{1}$ and its sub-structures are also nested sub-structures of community $C_{8}$ as can been seen in Fig. 14.

Full investigation of a large social network is a major task. In practice, the analysis is started from the most important communities detected from the network. This has been the idea in Figs. 13, 14. Alternatively the analysis is focused on communities, or nodes, of special interest. Different search criteria for the analysed results can be used, for example, node numbers, community identifiers and values of community measures. Nested structures are discovered by comparing the compositions (nodes) of the detected communities. Deep nested hierarchies can exist when some sub-communities extend their influence widely, like $C_{1}$ in Figs. 12 and 14.

**Fig. 15:**
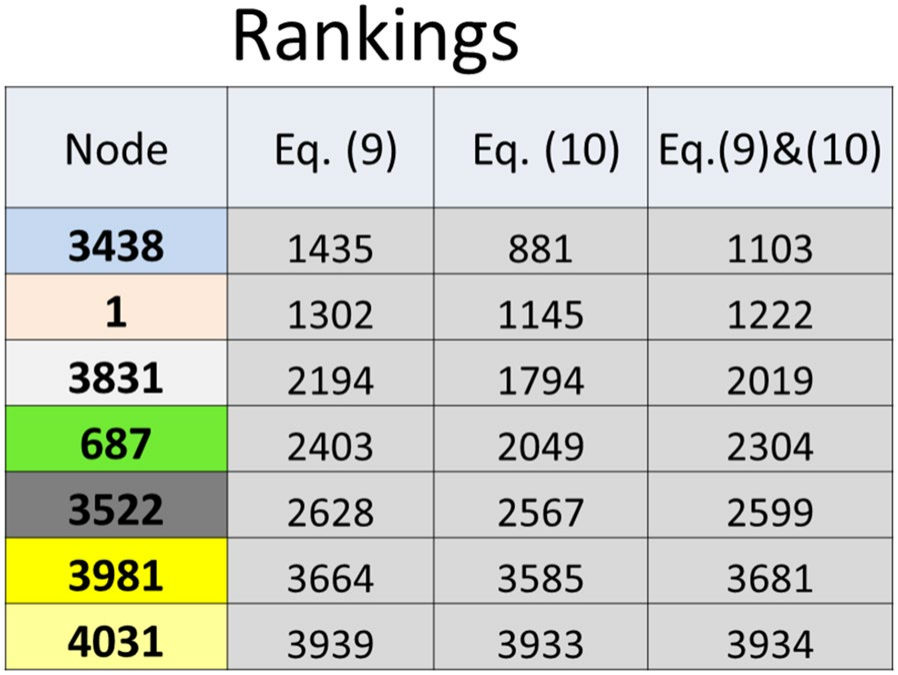
Nodes and their rankings with the highest closeness centrality values in sub-communities of Fig. 14. The last column shows the rankings of the average value of Eqs. (9) and (10)

Closeness centrality rankings of the most important nodes in sub-communities of Fig. 14 are shown in Fig. 15. Rankings of measures of Eq. (9) and (10) are not the same. However, their numerical values typically are close to each other.

## Conclusion

We consider a model with one ego initiating the influence spreading process in a social network. This allows us to study different phenomena in structured networks. In practical calculations, the proposed model can also be used for simultaneous source nodes of influence spreading. Dynamic measures for spreading in a social network are used as measures for centrality and betweenness. These measures are functions of network activity and time. Time can be interpreted as the development phase of social relations in a social network. A steady state is reached at high values of time. Therefore, measures describing centrality, betweenness or other characteristics of a network, can be calculated for the steady state or for different development phases of a network.

The proposed model takes into account different paths of the network from a source node to target nodes. Secondly, the dependency of paths is modelled by considering common links at the beginning of the paths. Combining these aspects is the novelty of the model compared to other models in the literature. These features of the model enable many opportunities to study new phenomena in complex networks and to solve existing problems more accurately.

Highly connected peripheral groups have multiple possible paths at the beginning of the spreading process which emphasizes the importance of these nodes as influential spreaders. It is known that initial spreading dynamics is crucial for later development of dynamic processes in a network [47].

We consider networks with a constant structure of nodes and links. Influence is spreading in the network and one node can spread similar influence repeatedly. Results in several recent articles [48–50] indicate that peripheral nodes, which are not highly influential, have more spreading power than most of the existing models predict. Our study provides evidence supporting these statements. Allowing loops (a node is allowed in the same chain of links more than once) further enhance the importance of interconnected peripheral nodes with low connectivity to central core network structures.

Activity of nodes has a nonlinear effect on rankings of the most influential nodes in the network. For example, if nodes’ activity is lower, the prominence of peripheral connected nodes is higher. We can say that the activity of nodes is an important aspect and models should take activity as one of the main variables of dynamic social network analysis and influence measures. In the model, activity is described by node and link weighting factors.

A new community detection measure is proposed in this paper. The community detection algorithm can be used to analyse possible sub-communities or closely connected members of the network. The idea in analysing community structures is based on the concept of nodes’ role in the network as sources and targets of influence. Both of these aspects have a role in community formation. The algorithm computes local maxima of an influence measure which considers both in- and out-directions of influence. Typically, social networks with weak interactions between nodes or social networks that are at their early development phases have several local maxima with different compositions. These factions can intersect and overlap with each other.

In this paper, we propose a consistent modelling framework for computing powerful influence spreaders and mediators in a social network. The same theory can be used in analysing community structures. The method is discussed and illustrated with several examples and graphical presentations.
